# Creutzfeldt-Jakob Disease as an Ongoing Concern: A Narrative Review

**DOI:** 10.7759/cureus.117478

**Published:** 2026-10-05

**Authors:** Leah R Helm, Alexander R Toftness

**Affiliations:** 1 Neuroscience, Coe College, Cedar Rapids, USA

**Keywords:** cjd pathophysiology, creutzfeldt-jakob disease, neurodegeneration, prion diseases, prnp gene, protein misfolding, tauopathies, transmissible spongiform encephalopathies

## Abstract

Creutzfeldt-Jakob disease (CJD) is a rare neurodegenerative disorder that makes up the majority of cases of prion disease. It is relatively rare, with a variety of subtypes that have significant variation in phenotype and etiology. Further, CJD shares a close relationship with other neurodegenerative diseases and is often underreported due to notorious difficulties in diagnosis. Recent developments, however, have improved the likelihood of clinical diagnosis. Additionally, there have been a number of research efforts in recent years toward CJD treatments. These recent developments and the potentially great benefit of progress in CJD research have spurred the creation of this narrative review to provide an overview of the current research on CJD and related prion diseases. Areas of focus include CJD subtypes, related prion diseases, epidemiology, pathophysiology, presentation/diagnosis, treatments, and, finally, connections with other diseases. Efforts were made to create an accessible review that could be useful as a precursor to future research.

## Introduction and background

Creutzfeldt-Jakob disease (CJD) is a rare neurodegenerative disorder. It is categorized as a transmissible spongiform encephalopathy (TSE), also known as a prion disease. CJD was first reported independently by Drs. Hans Creutzfeldt and Alfon Jakob in 1920 and 1921, respectively. While there is some debate as to whether all of the cases reported by the two doctors meet the modern CJD diagnostic criteria, they still constitute the earliest reported cases [1]. CJD being caused by a unique pathogen, misfolded proteins, was reported in 1982 by Stanley Prusiner, who won a Nobel Prize for the work [2]. Understanding of that unique pathogen has developed since then but remains essentially true. CJD results from the presence and activity of the pathogenic isoform of the normal cellular prion protein, PrP^C^, known as either PrP^S^^c^ (scrapie), PrP^dis^ (disease) [3], PrP^res^ (resistant to proteases) [4], mfPrP (misfolded) [5], or PrP^TSE^ (transmissible spongiform encephalopathies) [6]. The specific PrP^TSE^ responsible for CJD is notable for its protease resistance and resulting clinical presentation. Misfolded proteins are also associated with non-prion diseases including Alzheimer’s disease, Parkinson’s disease and chronic traumatic encephalopathy (CTE) [7,8]. These are related to prion disease and, along with them, can be called amyloidogenic diseases. CJD is invariably fatal, typically within a year after clinical signs develop, with disease durations varying between subtypes [9]. CJD is estimated to affect 1-2 people per million annually and is more common later in life, typically at ages 55-75 [10,11]. There is a notable lack of treatments for CJD, and while there are many efforts underway, none have progressed past cautious and limited patient trials. 

Considering the current progress in emerging therapies and the relationship CJD has with other neurodegenerative diseases, breakthroughs in CJD research could prove to be both possible and incredibly valuable. Several aspects of current knowledge of the nature of CJD, as well as emerging research developments, will be explored here. This review seeks to provide a useful and accessible overview of several aspects of current research into CJD to guide future research.

## Review

Methods

Encouraged by recent developments in therapies and diagnostic tools for CJD, we opted to create a broad picture of the current state of CJD research and its place relative to TSEs by analyzing a variety of peer-reviewed sources with an emphasis on original research. Given the focus on qualitative synthesis of information, no quantitative meta-analysis was conducted. The majority of sources were found using Google Scholar along with works referenced therein. A focus was placed on research from 2022 to 2026, although older resources were used to assist in informing the foundation and context of the newer research. Priority was given to high-impact, relevant work, and sources were chosen so as to support our goal of creating an interconnected and comprehensible presentation of the research. 

Subtypes

CJD is generally recognized in three categories: genetic, sporadic, and acquired, the latter of which includes the subcategories of iatrogenic and variant.

Genetic CJD

Genetic CJD (gCJD or fCJD for familial) is similar to other genetic prion diseases, such as Gerstmann-Sträussler-Scheinker (GSS) disease and fatal familial insomnia (FFI). In fact, all of the genetic prion diseases share the etiology of mutations in a single gene, the PRNP gene, although they have unique enough characteristics to be separately categorized. GSS is an autosomal dominant condition found in a handful of families, generally all with a mutation, possibly unique to that family, found somewhere in the PRNP gene [12]. It is characterized by PrP^TSE^ plaques, p-tau deposits, and neurofibrillary tangles made of tau aggregates, the latter two of which are also found in Alzheimer’s disease and CTE, earning them all the label of tauopathies [13,14]. GSS mostly presents as ataxia, spastic paraparesis, and eventual cognitive decline [12], but phenotypes vary among specific mutations. FFI is similar in genetic etiology but is noted for presenting most often with persistent insomnia as an initial symptom. It then progresses into physical and cognitive symptoms not unlike CJD, such as rapidly progressive dementia [15]. Sporadic fatal insomnia cases that are not linked to genetics have also been reported, but they are very rare and may be considered the thalamic version of the MM2 variant of sCJD [16]. Similar to other genetic prion diseases, gCJD varies in presentation based on the specific mutation at hand. A variety of mutations have been reported, including E200K, D178N (associated with FFI), P102L (associated with GSS), V210I, Q160K, and T188K (Table 1). However, the E200K mutation is most common and has the largest body of research [17,18]. With the addition of the next most common D178N and P102L varieties, the three account for an estimated 53% of genetic prion cases [19]. gCJD in its typical form is similar to classical CJD, with symptoms including cognitive (e.g., dementia), cerebellar (e.g., ataxia), and sensory disturbances.

**Table 1:** Mutations Associated With Genetic CJD and Their Presentations, Features, and Progression gCJD: Genetic Creutzfeldt-Jakob disease; t-tau: total tau; EEG: electroencephalogram; CJD: Creutzfeldt-Jakob disease.

Mutation	Presentation and features	Progression	Sources
E200K	Standard gCJD	2–41 months	[17]
T188K	Common in China, homozygous mutation	5 months	[20,21]
V180I	More likely abnormal (negative) results on 14-3-3, t-tau, EEG. Less clinical signs than other forms	Longer disease duration	[22]
V210I	Less penetrant than other forms, approximately 10% lifetime risk. Often involves visual symptoms	Shorter disease duration	[19,23]

Sporadic CJD

The majority of CJD cases are sporadic, meaning that the presence of PrP^TSE^ in the brain has a currently unexplained cause. Genetics have an impact on sporadic (and iatrogenic) cases but do not serve as a comprehensive explanation. The sequence at PRNP codon 129 has been shown to have major effects on the presentation and progression of the disease. Generally, the presence of a methionine at codon 129 of PRNP makes the structure of the resulting PrP^TSE^ more stable, and so those who are homozygous for methionine (129 MM) will see the most severe disease forms [24]. Further, it is somewhat rare for people who are homozygous for valine (129 VV) to develop the disease [5]. The reasons for this are yet unclear, but a significant factor may be the notable structural differences in the pathogenic protein isoform shown between the genotypes [24].

Even within sporadic CJD, there are many subtypes, generally classified based on two factors. As mentioned, one factor involves the amino acid present at codon 129 of the PRNP gene that codes for the prion protein, which appears to affect how prion disease develops. The other factor used in classification is the reaction of the protein to proteinase K cleavage and the resulting fragment sizes. This results in a core molecular mass of either ~21 kDa or ~19 kDa, corresponding to type one or type two, respectively [5,16]. While this subtyping system can be used with other forms such as genetic CJD, it is most often applied to the sporadic form [17]. Thus, there are theoretically six subtypes of sCJD, each with slightly different presentations and prognoses. Notably, the MM1 and MV1 genotypes are often grouped together, and the MM2 variant is typically divided into cortical and thalamic versions, which still results in six versions. The thalamic version of MM2 can be considered sporadic fatal insomnia on the basis of presentation, despite the different categories [6]. Overall, the number of subtypes and heterogeneity between them make diagnosis remarkably challenging (Table 2).

**Table 2:** Subtypes of sCJD and Their Presentations, Features, Average Clinical Durations, and Frequencies ^1^A MV2 subtype with no kuru plaques exists and is often known as MV2(C), for its dominant cortical pathology. sCJD: sporadic Creutzfeldt-Jakob disease.

Type	Presentation and features	Average clinical duration	Frequency (% of sCJD cases)	Sources
MM(V)^1^	Classical CJD: spongiform, mostly in cortical layers	~4 months	60-70%	[5]
MM2C	Cortical variant. Large and coalescent vacuolations throughout the cortical regions, basal ganglia, and thalamus	~25 months	With MM2T, ~5% of cases	[25]
MM2T	Thalamic variant, also known as sporadic fatal insomnia. Mild spongiform change and patchy PrP^TSE^ accumulation	~23 months	With MM2C, ~5% of cases	[25]
VV1	Moderate to severe microvacuolation throughout the cerebral cortex	~21 months	~1%, rarest	[5]
VV2	Most severe vacuolation in cerebellum	~6 months	10-15%	[5]
MV2(K)^1^	Kuru-type amyloid plaques, solid rounded structures composed of a PrP^TSE^ core and radiating fibrils	17-18 months	~10%	[5]

Variant CJD

Acquired CJD comes in two forms: variant (vCJD) and iatrogenic (iCJD). Variant CJD occurs when bovine spongiform encephalopathy (BSE), known more colloquially as “mad cow disease,” spreads to humans. The outbreak of BSE in the UK during the 1980s and 1990s resulted in more publicity for prions, and ongoing wariness has resulted in continued precautions despite the very low likelihood of a return of the epidemic [26]. Most of these precautions were centered around the processing and consumption of beef products, given that the majority of cases likely resulted from ingestion of infected beef. Concerningly, there have been reported cases of CJD being acquired after a blood transfusion from someone with vCJD (such cases can be designated vCJD or iCJD). However, this resulted in most of England putting blood products through leukodepletion before transfusion, and no further cases have appeared via this transmission route since that process began in 1999 [26]. The process of leukodepletion was likely successful due to the role of the immune system in the transmission of vCJD. Many immune cells are capable of expressing the prion protein, and so exposure to leukocytes containing PrP^TSE^ has been suggested to transmit vCJD into the host’s immune system and from there into the brain [27]. This is supported by findings that even preclinical vCJD cases saw buildup of PrP^TSE^ in immune tissue such as the spleen, appendix, and lymph nodes [28,29]. It is worth noting that there were only ever about five cases of vCJD secondary to transfusion with vCJD-implicated blood products, and thus, given the time elapsed and lack of further cases, some precautions are being reduced. This includes discontinuing donor deferral for those potentially exposed (by geographical location during a certain time period) to help avoid unduly reducing the blood supply [30]. Further, two atypical forms of BSE have been noted without transmission to humans but have been shown to be potentially infectious in animal experiments. These are L-BSE and H-BSE, named for the lower and higher molecular weights of the PrP^TSE^, respectively, as compared to classic BSE. While H-BSE has been shown to have longer disease durations and to be generally less transmissible, L-BSE has been shown to be more transmissible than classic BSE, further recommending continued caution around prions in livestock [4,31]. Additionally, it is hypothesized that L-BSE could be the origin of several transmissible mink encephalopathy outbreaks [31].

Iatrogenic CJD

Iatrogenic CJD is the rarest subtype of CJD. It is associated with cases in which the physical prion protein entered an individual’s brain and resulted in the pathology. This route of transmission can result from direct contact with the brain via transplants or surgical materials or, less directly, via the immune system [27]. However, due to the rarity of CJD, there are very few of these cases. Some examples include the use of cadaver-derived CJD-contaminated hormones: human growth hormone (c-hGH) or gonadotropin. Additionally implicated in the transmission of iCJD are dura mater transplants, corneal transplants, depth electrodes (for stereotactic EEGs), and the use of contaminated surgical equipment in neurosurgery [32]. Further, the c-hGH implicated in spreading iCJD has also been associated with iatrogenic Alzheimer's disease. The pooling methods used with the hormones before their use in patients mean that the proteins capable of seeding both diseases were present, albeit at low concentrations, in significant portions of the utilized hormone [33,34]. These results in relation to Alzheimer's disease have been criticized on the basis of insufficient evidence of tau pathology in the affected patients, preventing a convincing diagnosis of clinical Alzheimer’s disease [35]. However, this could be due to iatrogenic Alzheimer’s disease having a distinct phenotype from the classic sporadic or inherited forms [36].

The primary reason that iCJD was transmitted via neurosurgery and EEG is that prions are not effectively denatured by standard decontamination procedures. In fact, allowing contaminated equipment to dry, as occurs during heating, increases prion stability [37]. To inactivate prions, current recommendations include the use of either sodium hydroxide or sodium hypochlorite in addition to an autoclave with temperature settings above typical heat standards, although it is ideal to avoid the need for such extreme inactivation measures through the use of disposable tools and coverings that can be incinerated after use [38]. Iatrogenic CJD generally has a comparatively short incubation time and therefore an earlier age of onset than other types, with average incubation periods as low as 1.5 years in the case of depth electrodes and up to 17 years in the case of c-hGH [37].

Epidemiology

While a rarity when placed among all diseases, CJD is one of the most common of the group of prion diseases, allowing sufficient investigation of its epidemiological characteristics. The occurrence of CJD is even between sexes, and typical onset is from 55-75 years of age, although some subtypes, such as vCJD, are seen earlier in life on average [10,39]. Sporadic CJD is the most common form of not only CJD but prion diseases as a whole, accounting for about 88% of cases [11]. Genetic prion diseases of all kinds, including gCJD as well as GSS and FFI, together account for around 10-15% of cases. In contrast, acquired cases represent remarkably small percentages. There are some countries in which the genetic percentages are much higher, which stands to reason given the likelihood of common genetics among people in a shared area. For example, Israel and Slovakia both have percentages of genetic prion disease (as compared to prion disease as a whole) around 60% as well as overall higher incidences of prion disease, so the greater genetic proportion does not solely indicate a lower incidence of other forms [11]. Worldwide, CJD typically affects around 1-2 people per million per year. This number must be considered in light of the fact that CJD is often underdiagnosed and underreported. There are not many specific tests for CJD, and those that exist are not widely available or implemented. In many cases, the only real way to gain confirmation is a brain biopsy, which is unlikely to be widely performed. Additionally, there are many more common disorders for which CJD may be mistaken. The lack of treatments for CJD also means that more treatable disorders will be ruled out first, thus delaying and, given the typical prognosis, preventing premortem CJD diagnosis. There are reports from several countries specifically discussing the likelihood of underdiagnosis of CJD in their area; these include Thailand and Vietnam [40,41].

One concern with regard to the epidemiology of CJD is that the number of cases increased over time, at least across the period from 1993 to 2020 [11]. The effect of increased reporting and diagnostic ability, as well as a generally aging population (considering the long incubation periods), on this trend cannot be discounted. There is, however, a possibility that as time passes, there will be an increase in mutations with the potential to cause CJD (and other prion diseases, such as GSS) due merely to random chance. These will subsequently be passed down, slowly but significantly increasing the total number of CJD cases. Regardless of cause, the increasing number of CJD cases does represent an area of concern.

Pathophysiology**

The gene for the prion protein, PRNP, is expressed in many parts of the body, predominantly in the neurons and glial cells of the brain, but also in some parts of the immune system. The role of this protein in its typical cellular function is not entirely clear. It has been suggested that it may play roles in cell adhesion, copper metabolism, or nucleic acid transport, as well as having interactions with many membrane receptors [39]. PrP^C^, however, is likely not essential enough for the loss of that function to be a major contributing factor to the disease [42]. PrP^C^ is membrane-bound with a structured globular domain and an unstructured flexible tail and so can be exposed to PrP^TSE^ without the misfolded protein needing to enter the cell [43]. Once that PrP^C^ protein has been exposed to PrP^TSE^, its form undergoes structural modification, primarily including a greater proportion of beta-pleated sheets, but there are subtler structural changes, such as the induction of an H-latch formation, which has a role in prion toxicity [43]. CJD has several pathogenic effects in the brain. The most distinctive are neuronal death and spongiform patterning that occur due to the disruption of intracellular proteins via alteration to their folding, ubiquitination, and trafficking within neurons, primarily in the gray matter [44]. Causes of neuronal death, such as apoptosis via multiple mechanisms, including disulfidoptosis, have also been noted [45]. Additionally, it has been suggested that an anti-PrP^TSE^ antibody, specifically POM1, may have neurotoxic effects in and of itself [43]. Other pathogenic effects include an increased presence of highly reactive astrocytes and microglia [39,45]. Astrocytes, despite their increased protective role in clearing both cellular debris and apoptotic bodies during the CJD course, nevertheless are seen to have a net negative effect via their role in damaging synaptic integrity and lowering dendritic spine size and density [46].

Presentation and diagnosis of CJD

CJD, in any given form, has a variable and remarkably nonspecific presentation. Most of even the classic symptoms are shared with many other neurodegenerative diseases. The standard presentation includes rapidly progressive dementia, myoclonus, parkinsonism, aphasia, pyramidal and extrapyramidal signs, and even akinetic mutism toward the end of the disease progression [47]. It is difficult to distinguish CJD from conditions such as Alzheimer’s and Parkinson’s, which are related in mechanism as well as similar in presentation. Historically, it was difficult, if not impossible, to diagnose CJD before death, resulting in many diagnoses of possible or probable CJD (Figure 1). 

**Figure 1:**
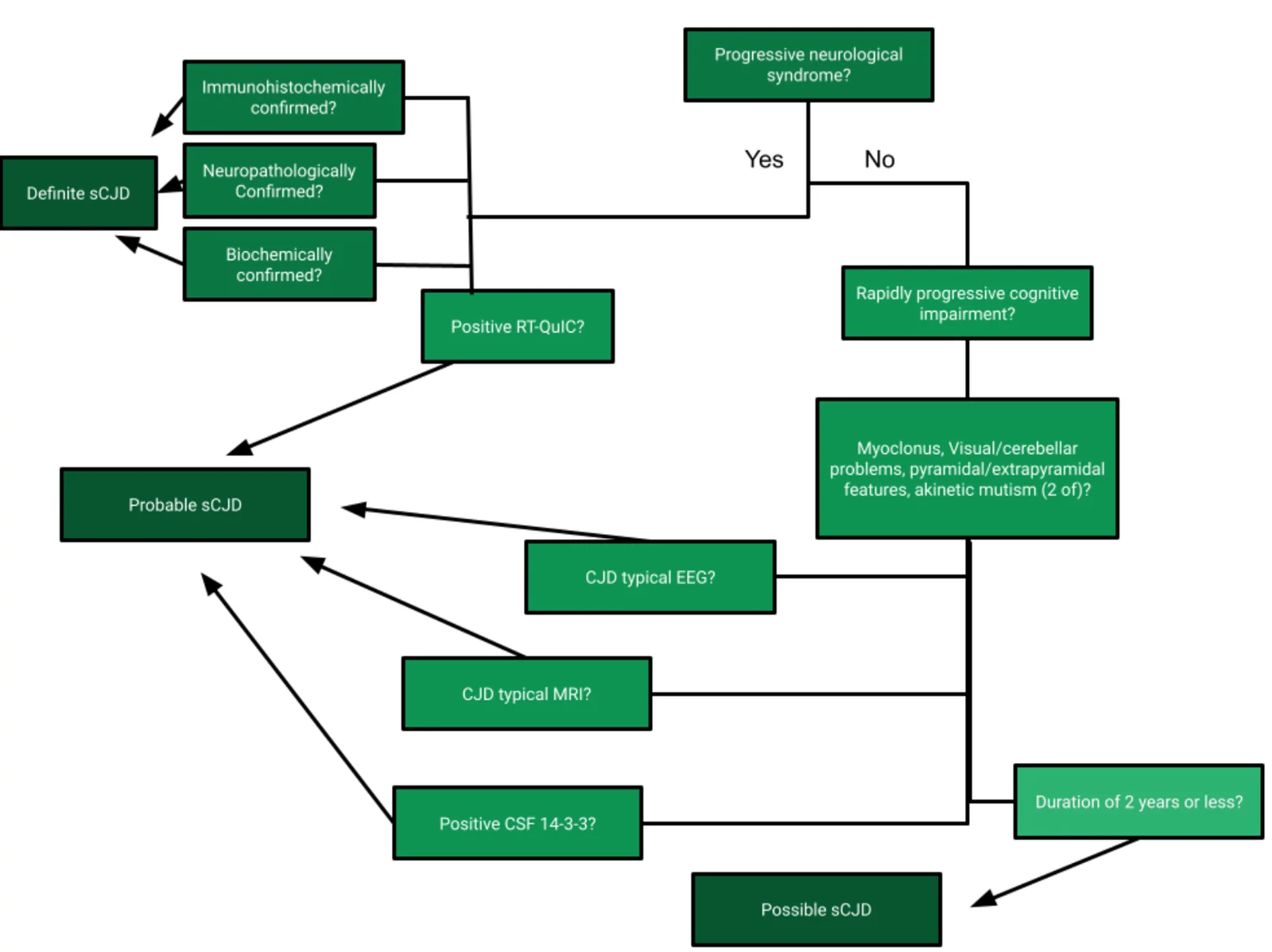
Representation of Diagnostic Criteria for sCJD Criteria from the International CJD Surveillance Network [48]. sCJD: sporadic Creutzfeldt-Jakob disease; RT-QuIC: real-time quaking-induced conversion; EEG: electroencephalogram; MRI: magnetic resonance imaging.

Biomarkers

The most reliable diagnostic method remains direct analysis of brain tissue, but recent improvements in biomarker research have made earlier diagnosis possible. Some examples of biomarkers include real-time quaking-induced conversion (RT-QuIC) and 14-3-3 proteins, both of which can be observed in cerebrospinal fluid after a spinal tap. RT-QuIC takes advantage of PrP^TSE^’s ability to form PrP^C^ into fibrils that can bind to a fluorescent dye, making them identifiable. RT-QuIC is one of the most specific tests available and can detect trace amounts of PrP^TSE^ in CSF [49]. The addition of RT-QuIC greatly improved the sensitivity of diagnostic criteria [50]. There is also the theoretical ability to identify RT-QuIC in blood; however, this generally has less specific and sensitive results than CSF analysis. Additionally, most of these tests are specific to variant CJD [51]. Detection of 14-3-3 proteins is generally done with western blots or enzyme-linked immunosorbent assay (ELISA) technology, with 14-3-3γ ELISA having sensitivity between 80% and 90% and specificity between 70% and 92% [52]. 14-3-3 proteins are present in response to rapid neuronal death in general, and so the specificity is necessarily imperfect. An additional biomarker used is tau protein, and tau/p-tau ratios are used to distinguish CJD from Alzheimer’s disease. In CJD, the tau/p-tau ratio is much higher than in Alzheimer’s disease due to the higher quantities of p-tau present with Alzheimer’s disease. The proposed cutoff for CJD is a ratio of tau to p-tau of either 18:1 or 20:1 depending on the testing method [53].

Imaging

Imaging is also a valuable addition to diagnostic methods. MRI and EEG are both often utilized and are useful for both identifying CJD and ruling out other possible explanations for symptoms.

Diffusion-weighted MRI (DWI) and fluid-attenuated inversion recovery (FLAIR) techniques are both often used to reveal CJD-associated abnormalities, although DWI tends to be slightly more sensitive. A common MRI finding is signal abnormalities in many cortical regions, including the frontal lobe. The cerebellum is only occasionally implicated, showing signal hyperintensities typically in sCJD cases with the VV2 type [54]. Additionally associated with CJD are hyperintensities and associated restricted diffusion in the bilateral caudate heads and putamen [40,55,56]. The pulvinar and “hockey-stick” signs are more common in vCJD but have been observed in other forms [54]. Cortical ribboning is also a common sign and is typically more visible on DWI than FLAIR [57]. Cortical ribboning has also been noted to be more apparent early in disease progression than other signs [51]. There have been some instances of patients with confirmed (e.g., via brain biopsy) CJD who had unremarkable MRI results during the course of their illness [54,55]. While this could be due to some degree of error in noting subtle signs, the sensitivity and specificity of MRI for CJD remain imperfect [55]. MRI does have a better ability to detect early CJD indications, however, as compared to EEG and CSF markers, which often only return significant results later in the course of the illness [39,51].

The distinctive electroencephalography (EEG) sign is periodic sharp wave complex (PSWC) patterns [17]. The PSWC is markedly indicative of CJD cases, but not all CJD cases will display them, especially early in disease progression. For sCJD, PSWC can be simple or complex and generally recurs every 0.5-2 seconds with a duration of 100-600 ms. The PSWC is typically entirely missing in vCJD cases [51].

Emerging therapies

In the past few years, there have been a number of ongoing research efforts for CJD treatments. Few of these include clinical trials, limited significantly by the rarity and rapid progression of the illness; however, animal and in vivo studies are not uncommon. Given that the cellular form of the prion protein does not contribute to neurodegeneration by its absence alone, there are some treatments that target the normal cellular protein before it has had the chance to become harmful. One example of this is a trial with six CJD patients to test a monoclonal antibody, PRN100, targeting the normal cellular prion protein. Disease progression was halted for the duration of treatment in 75% of the patients in whom the therapeutic dose was reached, but neurodegeneration was not reversed in any of the cases. Further, the researchers noted no adverse side effects from the treatment [58]. This is not the only immunotherapy currently used in CJD trials; strategies generally consist of antibodies and vaccines via passive and active immunotherapy. Three CJD-specific epitopes have been used to prevent and control prion protein growth in conjunction with PRNP knockout [59].

One further treatment is the class of antisense oligonucleotides (ASOs), which are utilized for PRNP knockdown and the reduction of the quantity of PrP^C^. A particular ASO known as ION717 is currently in clinical trials and works to support PRNP mRNA degradation to reduce the expression of PrP^C^ and therefore the prion pathology [60,61]. It has seen tentative early successes in terms of the interaction with the target protein as well as in safety and tolerability [62]. These trials come after other effective ASO treatments for non-prion neurodegenerative conditions [61].

Other potential treatments include a variety of chemicals, including Congo red, dimethyl sulfoxide, ethanolamine, dextran sulfate, and doxycycline [59]. Beyond that, RNA treatments using shRNA and siRNA have been shown in animal models to reduce the activation of PrP^C^ mRNA and therefore the expression of PrP^C^ and accumulation of PrP^TSE^. Additionally, using lentivirally mediated RNAi, PrP^C^ knockdown was shown to reduce PrP^TSE^ accumulation [63]. Given the beta-sheet-heavy structure of PrP^TSE^ as opposed to PrP^C^, some treatments have been based on interference with beta sheets. One of the more prominent advances here is a family of peptides known as beta-sheet breakers. Specifically, a modified cyclic β-sheet peptide has been shown to have anti-amyloidogenic effects, both in inhibiting aggregation and disrupting existing aggregations [64].

Additionally, there are methods that use stem cells. Mesenchymal stem cells have been shown to be protective against prions by releasing neurotrophic factors near lesions. Additionally, neural stem cells can potentially replace damaged or killed neurons and glia. Overall, some of these have made it into clinical trials, although blood-brain barrier penetration and challenges with early diagnosis were significant enough problems to prevent tangible results in most attempts [59]. Clinical trials with CJD are difficult, given the limited patient pools and time restrictions. However, many treatments have seen success in animal models, and therefore human treatments may be within reach.

One additional consideration is that prions do seem to have the ability to develop drug resistance via strain selection. That is, a given group of PrP^TSE^ has a variety of specific forms of PrP^TSE^, which means that if a drug is more effective against one variety than another, then it is possible that the unaffected strains will propagate further. This means that a variety of drugs may eventually be required to provide effective treatment, which is a concern when we currently lack a single genuinely effective drug [65]. This is not an area with much research as of now, but it may be worth considering.

Connections with other diseases

Further, despite the uncommon pathology of CJD, it has commonalities and connections with a number of other conditions. 

Related Prion Diseases

While CJD is the most common prion disease, it is not the only one. Kuru is another human TSE. Kuru was one of the first human prion diseases to be recognized and is currently regarded as extinct. It was first reported in the 1950s among the Fore people of Papua New Guinea, although it appeared to have existed for a significant period previously. It is generally attributed to the practice of ritual cannibalism, which was conducted as a gesture of respect for the dead and involved consumption of human brain tissue, which turned out to be infected with prion protein. Kuru presented with a variety of symptoms, ataxia primarily among them, but vision disturbances, photophobia, abnormal or absent reflexes, hypotonia, and emotional instability (e.g., uncontrolled laughter) were also common. The general life expectancy was about 8 months after the beginning of clinical signs, though the incubation period could be up to 56 years [66]. 

VPSPr is another sporadic human prion disease, recently reported in 2008. It differs from sCJD in disease presentation, the form of the PrP^TSE^ and the resistance of the PrP^TSE^ to proteinases. Typical CJD-associated PrP^TSE ^are resistant to cleavage by proteinases, but when proteolysis by PK occurs on VPSPr-associated isoforms, at least three fragments of PrP^TSE^ result, indicating VPSPr’s distinction from sCJD [67]. VPSPr has less resistance overall; however, there is still phenotypic variation. It shares sCJD’s variability based on the gene at codon 129, but is somewhat inverted, appearing to be most common in those with the 129VV genotype. Further, in many aspects, VPSPr more closely resembles GSS than CJD [3,68]. 

Among animals, there have been many reported cases of prion diseases. The earliest is scrapie, first described in England in 1732. Scrapie is found in sheep and goats and it is possible that BSE originated due to scrapie jumping into cows and from there into humans, then being known as vCJD (as previously discussed) [69]. Scrapie has both a classic and atypical form, the former being generally iatrogenic and the latter sporadic. Classic scrapie is transmitted mostly orally, although many other routes have been shown to result in transmission. There is greater involvement of the immune system with scrapie; PrP^Sc^ accumulation is seen in lymphoid tissues, especially gut-associated lymphoid tissues (GALTs). This means that pre-symptomatic diagnosis of scrapie is possible [70]. 

Cervids, such as deer, moose, and elk, have been seen to experience Chronic Wasting Disease (CWD). CWD has not been shown to be transmissible to humans, but has been transmitted experimentally in non-human primates [2], and in vitro experiments have shown that PrP^CWD^ does have the capability to convert human PrP^C^ [71]. Therefore, precautions are nonetheless in order. One significant concern with CWD is that the associated prions have been shown to be able to bind to soil and be taken into plants. That fact, in combination with the frequent shedding of PrP^CWD^ into the environment by infected animals, means that large portions of a given population will likely develop CWD [2]. This further increases the likelihood of human exposure and thus it would be valuable to have a better understanding of what has prevented confirmed human cases, along with the potential ability to recreate that effect with other prion diseases or among uninfected cervids. 

Other Neurodegenerative Diseases

The pathology of both Alzheimer’s disease and Parkinson’s disease is often considered to be associated with particular forms of misfolded proteins, a somewhat similar etiology to prions. The protein in question differs, as these are associated with amyloid-beta plaques and the deposition of neurofibrillary tangles of phosphorylated tau for Alzheimer's and α-synuclein aggregates, pale bodies, and Lewy bodies for Parkinson’s. Like CJD, Parkinson’s disease has both genetic and sporadic forms, though genetics influence the qualities of the sporadic form [72]. Both of these other neurodegenerative diseases have been shown in animal models to be potentially transmissible, and there have been reports of iatrogenic Alzheimer’s disease, particularly due to some of the same modes of infection as vCJD. As previously discussed, this primarily includes cadaver-derived pituitary growth hormone (c-hGH) [33]. While not strictly the same as CJD, a certain type of the related prion disease, GSS, has been found to show p-tau fibrils that are apparently identical to those found in Alzheimer’s disease [13]. 

These connections provide further reasoning for investing resources into researching CJD and prion diseases in general, given that any advances here could reasonably be expected to offer insights into conditions like Alzheimer’s disease and Parkinson’s disease, both of which are dramatically more common and typically considered more impactful than the ultimately rare CJD. 

Potential COVID-19 Association

The neurological alterations accompanying coronavirus disease 2019 (COVID-19), as caused by SARS-CoV-2, have been a point of interest, especially given its extremely long-lasting variations. There have been at least 20 case studies in recent years of CJD developing after or during an infection with COVID-19 [10,73-92]. Evidence is limited as to causality, but a plausible interaction between the pathogens seems supported by other neurological symptoms of “long COVID.” The theory is that COVID-19 may not specifically create CJD, but accelerate the progression of a disease already in its early stages [73]. The connection is compelling given the unusual features of CJD following COVID-19 infection (a high age of onset and especially rapid progression), but nothing beyond a temporal association can be confidently stated [10,74].

## Conclusions

Despite the condition’s rarity, CJD is a condition for which progress towards a treatment could be pointedly valuable. The overall lack of scientific understanding of the wide varieties of TSEs, exemplified by the uncertain etiology of sCJD, indicates that there is much research left to do. Further, the presence of promising, if not perfect, experimental treatments indicates the existence of a forward path. Not merely forward for CJD, or even prion diseases as a collective, but neurodegenerative diseases on the whole. The close relationship that CJD shares with other amyloidogenic conditions of the brain means that progress in one could offer progress in others. The similar etiologies and, at times, methods of transmission suggest similarities that could readily translate into shared research goals and achievements. With that, the effects of progress in CJD treatments could be as wide-reaching as the hundreds of millions of people affected by Alzheimer's disease. Further, recent progress in identifying CJD pre-mortem, via biomarkers and imaging, and the continual discovery of genetic indicators both mean that the identification of patients before onset or early in disease progression is more possible than it once was. This both allows for better patient care and gives clinical trials and treatments more time to be effective, bolstering research efforts. Therefore, it is both crucial and possible that progress continues.

## References

[1] Katscher F (1998). It's Jakob's disease, not Creutzfeldt's. Nature.

[2] Holman AP, Kurouski D (2025). Prion diseases: lessons from historical outbreaks and potential emerging ones. Protein Sci.

[3] Zou WQ, Puoti G, Xiao X (2010). Variably protease-sensitive prionopathy: a new sporadic disease of the prion protein. Ann Neurol.

[4] Mestre-Francés N, Nicot S, Rouland S (2012). Oral transmission of L-type bovine spongiform encephalopathy in primate model. Emerg Infect Dis.

[5] Ritchie DL, Smith C (2025). Pathological spectrum of sporadic Creutzfeldt-Jakob disease. Pathology.

[6] Saitoh Y, Mizusawa H (2024). Prion diseases, always a threat?. J Neurol Sci.

[7] Stein TD, Montenigro PH, Alvarez VE (2015). Beta-amyloid deposition in chronic traumatic encephalopathy. Acta Neuropathol.

[8] Alraawi Z, Banerjee N, Mohanty S, Kumar TK (2022). Amyloidogenesis: what do we know so far?. Int J Mol Sci.

[9] Price JR, Kheirbek RE (2024). Addressing the unmet needs of patients with rapidly progressive neurological disease: a case report of palliative care in Creutzfeldt-Jakob disease (CJD). Cureus.

[10] Begum A, Boppana MS, Rajavasireddy NS, Tummala N, Solís Mayorga MB (2024). Unusually late onset of Creutzfeldt-Jakob disease following COVID-19 infection in India: a case report. Cureus.

[11] Gao LP, Tian TT, Xiao K (2024). Updated global epidemiology atlas of human prion diseases. Front Public Health.

[12] Ribosa-Nogué R, Pagonabarraga J, Gomez-Anson B, Granell-Moreno E, Sánchez-Valle R, Kulisevsky J (2016). Gerstmann-Sträussler-Scheinker disease presenting with atypical parkinsonism, but typical magnetic resonance imaging findings of prion disease. Mov Disord Clin Pract.

[13] Bruno R, Pirisinu L, Riccardi G (2022). Gerstmann-Sträussler-Scheinker disease with F198S mutation induces independent Tau and prion protein pathologies in bank voles. Biomolecules.

[14] Taghdiri F, Khodadadi M, Sadia N (2024). Unusual combinations of neurodegenerative pathologies with chronic traumatic encephalopathy (CTE) complicates clinical prediction of CTE. Eur J Neurol.

[15] Qiao M, Wu H, Chi L (2024). A fatal familial insomnia patient initially misdiagnosed as Alzheimer's disease: a case report. BMC Neurol.

[16] Sun Q, Shen P, Tang J, Meng H, Feng J, Wang Z, Cui L (2021). Sporadic Creutzfeldt-Jakob disease appears to be sporadic fatal insomnia: a case report and review of the literature. Nat Sci Sleep.

[17] Appleby BS, Manca M, Piazza MS (2026). Genetic Creutzfeldt-Jakob disease linked to the E200K mutation: a large cohort study. Acta Neuropathol.

[18] Bregman N, Shiner T, Kavé G (2023). The natural history study of preclinical genetic Creutzfeldt-Jakob disease (CJD): a prospective longitudinal study protocol. BMC Neurol.

[19] Goldman JS, Vallabh SM (2022). Genetic counseling for prion disease: updates and best practices. Genet Med.

[20] Li CJ, Liang DL, Gao LP (2026). Comprehensive characterization of 98 Chinese cases of genetic Creutzfeldt-Jakob disease with T188k mutation. Ann Clin Transl Neurol.

[21] Shan Y, Zhang J, Cen Y, Xu X, Tan R, Zhao J, Yu S (2022). Creutzfeldt-Jakob disease associated with a T188K homozygous mutation in the prion protein gene: a case report and review of the literature. Prion.

[22] Nagata T, Shinagawa S, Kobayashi N, Kondo K, Shigeta M (2022). A case of V180I genetic mutation Creutzfeldt Jakob disease (CJD) with delusional misidentification as an initial symptom. Prion.

[23] Kong Y, Chen Z, Zhang J, Wang X, Wu L (2023). Clinical and genetic characteristics of the Heidenhain variant of Creutzfeldt-Jakob disease. Viruses.

[24] Omwansu W, Musembi R, Derese S (2025). Structural characterization of codon 129 polymorphism in prion peptide segments (PrP127-132) using the Markov State Models. J Mol Graph Model.

[25] Chen Z, Kong Y, Zhang J (2023). Toward an early clinical diagnosis of MM2-type sporadic Creutzfeldt-Jakob disease. Ann Clin Transl Neurol.

[26] Roberts B, Riley J, Evans TJ (2025). Estimating future variant Creutzfeldt-Jakob disease cases in the UK: a cohort-based probabilistic model. Lancet Reg Health Eur.

[27] Mabbott NA, MacPherson GG (2006). Prions and their lethal journey to the brain. Nat Rev Microbiol.

[28] Brown P, Cervenáková L, McShane LM, Barber P, Rubenstein R, Drohan WN (1999). Further studies of blood infectivity in an experimental model of transmissible spongiform encephalopathy, with an explanation of why blood components do not transmit Creutzfeldt-Jakob disease in humans. Transfusion.

[29] Llewelyn CA, Hewitt PE, Knight RS, Amar K, Cousens S, Mackenzie J, Will RG (2004). Possible transmission of variant Creutzfeldt-Jakob disease by blood transfusion. Lancet.

[30] Pozzo di Borgo A, Rochette S, Gaussen A, O'Brien SF, Germain M, Renaud C, Lewin A (2023). Transmission of variant Creutzfeldt-Jakob disease through blood transfusion and plasma-derived products: a narrative review of observed and modeled risks. Transfus Med Rev.

[31] Baron T, Vulin J, Biacabe AG, Lakhdar L, Verchere J, Torres JM, Bencsik A (2011). Emergence of classical BSE strain properties during serial passages of H-BSE in wild-type mice. PLoS One.

[32] Will RG (2003). Acquired prion disease: iatrogenic CJD, variant CJD, kuru. Br Med Bull.

[33] Banerjee G, Farmer SF, Hyare H (2024). Iatrogenic Alzheimer's disease in recipients of cadaveric pituitary-derived growth hormone. Nat Med.

[34] Purro SA, Farrow MA, Linehan J (2018). Transmission of amyloid-β protein pathology from cadaveric pituitary growth hormone. Nature.

[35] Nath A, Holtzman DM, Miller BL, Grinberg LT, Leschek EW (2024). Insufficient evidence for an association between iatrogenic Alzheimer's disease and cadaveric pituitary-derived growth hormone. Alzheimers Dement.

[36] Banerjee G, Mok TH, Hyare H, Cousins O, Jaunmuktane Z, Mead S, Collinge J (2026). High-level Alzheimer disease neuropathological change following iatrogenic exposure. JAMA Neurol.

[37] Bonda DJ, Manjila S, Mehndiratta P (2016). Human prion diseases: surgical lessons learned from iatrogenic prion transmission. Neurosurg Focus.

[38] (2026). CDC: clinical overview of Creutzfeldt-Jakob disease (CJD). CJD.

[39] Jurcau MC, Jurcau A, Diaconu RG, Hogea VO, Nunkoo VS (2024). A Systematic Review of Sporadic Creutzfeldt-Jakob Disease: Pathogenesis, Diagnosis, and Therapeutic Attempts. Neurol Int.

[40] Dumrikarnlert C, Kanokkawinwong N, Rattanabannakit C, Senanarong V (2024). Characteristics of Creutzfeldt-Jakob disease at Siriraj Hospital, Thailand: Case series and literature review. Clin Park Relat Disord.

[41] Vo KH, Bui NT, Hoang DT (2025). A descriptive analysis of sporadic Creutzfeldt-Jakob cases in Vietnam: 31 patients from four tertiary care centers. Clin Park Relat Disord.

[42] Mallucci GR, Ratté S, Asante EA, Linehan J, Gowland I, Jefferys JG, Collinge J (2002). Post-natal knockout of prion protein alters hippocampal CA1 properties, but does not result in neurodegeneration. EMBO J.

[43] Frontzek K, Bardelli M, Senatore A (2022). A conformational switch controlling the toxicity of the prion protein. Nat Struct Mol Biol.

[44] Hall WA, Masood W (2026). Creutzfeldt-Jakob disease. StatPearls [Internet].

[45] Wu Q, Liu SP, Liu C, Chen X, Zhou H, Zhao H (2024). Disulfidoptosis as a novel mechanism of neuronal death: insights from Creutzfeldt-Jakob disease. World Neurosurg.

[46] Makarava N, Kushwaha R, Baskakov IV (2024). Reactive astrocytes in prion diseases: friend or foe?. PLoS Pathog.

[47] Gurram S, Holla VV, Sharma P (2023). Spectrum and pattern of movement disorders in patients with sporadic Creutzfeldt-Jakob disease. Tremor Other Hyperkinet Mov (N Y).

[48] Watson N, Hermann P, Ladogana A (2022). Validation of revised International Creutzfeldt-Jakob disease surveillance network diagnostic criteria for sporadic Creutzfeldt-Jakob disease. JAMA Netw Open.

[49] Green AJ (2019). RT-QuIC: a new test for sporadic CJD. Pract Neurol.

[50] Mastrangelo A, Mammana A, Baiardi S (2023). Evaluation of the impact of CSF prion RT-QuIC and amended criteria on the clinical diagnosis of Creutzfeldt-Jakob disease: a 10-year study in Italy. J Neurol Neurosurg Psychiatry.

[51] Ramesh TS, Bartusik-Aebisher D, Dynarowicz K, Aebisher D (2026). Prion diseases-when proteins turn lethal: Creutzfeldt-Jakob disease (CJD) and the quest for classification, diagnosis, therapeutic approaches, and emerging research. Molecules.

[52] Shimamura MI, Satoh K (2025). Challenges and revisions in diagnostic criteria: advancing early detection of prion diseases. Int J Mol Sci.

[53] Fayolle M, Lehmann S, Delaby C (2022). Comparison of cerebrospinal fluid tau, ptau(181), synuclein, and 14-3-3 for the detection of Creutzfeldt-Jakob disease in clinical practice. J Neural Transm (Vienna).

[54] Manara R, Fragiacomo F, Ladogana A (2024). MRI abnormalities in Creutzfeldt-Jakob disease and other rapidly progressive dementia. J Neurol.

[55] Aslam S, Fritz MA, Cordes L, Sabbagh MN (2018). What promises the CJD diagnosis in a case of rapidly progressive dementia?. J Alzheimers Dis Parkinsonism.

[56] Salehi P, Clark M, Pinzon J, Patil A (2022). Sporadic Creutzfeldt-Jakob disease. Am J Emerg Med.

[57] Abdulmassih R, Min Z (2016). An ominous radiographic feature: cortical ribbon sign. Intern Emerg Med.

[58] Mead S, Khalili-Shirazi A, Potter C (2022). Prion protein monoclonal antibody (PRN100) therapy for Creutzfeldt-Jakob disease: evaluation of a first-in-human treatment programme. Lancet Neurol.

[59] Udayakumar S, Girigoswami A, Girigoswami K (2025). A review on current theories and potential therapies for prion diseases. Mol Biol Rep.

[60] (2026). A phase 1/2a study to evaluate the safety, tolerability, pharmacokinetics and pharmacodynamics of intrathecally administered Ion717 in patients with prion disease. https://clinicaltrials.gov/study/NCT06153966.

[61] Nikolić L, Legname G (2026). Multi-target approaches to prion disease drug discovery: a status update. Expert Opin Drug Discov.

[62] (2026). Community Statement to CJDISA announcing Regimen 3 added to PrProfile study. https://cjdisa.com/clinical-trial-update-for-prion-diseases/.

[63] Pfeifer A, Eigenbrod S, Al-Khadra S (2006). Lentivector-mediated RNAi efficiently suppresses prion protein and prolongs survival of scrapie-infected mice. J Clin Invest.

[64] Khairnar BD, Padhye A, Madiwal V (2023). Cyclic β-hairpin peptide loaded PLGA nanoparticles: a potential anti-amyloid therapeutic. Mater Today Commun.

[65] Ghaemmaghami S, Ahn M, Lessard P, Giles K, Legname G, DeArmond SJ, Prusiner SB (2009). Continuous quinacrine treatment results in the formation of drug-resistant prions. PLoS Pathog.

[66] Collinge J, Whitfield J, McKintosh E (2008). A clinical study of kuru patients with long incubation periods at the end of the epidemic in Papua New Guinea. Philos Trans R Soc Lond B Biol Sci.

[67] Imran M, Mahmood S (2011). An overview of human prion diseases. Virol J.

[68] Gambetti P, Puoti G, Zou WQ (2011). Variably protease-sensitive prionopathy: a novel disease of the prion protein. J Mol Neurosci.

[69] Betancor M, Marín B, Otero A (2023). Detection of classical BSE prions in asymptomatic cows after inoculation with atypical/Nor98 scrapie. Vet Res.

[70] Sola D, Betancor M, Marco Lorente PA (2023). Diagnosis in Scrapie: conventional methods and new biomarkers. Pathogens.

[71] Davenport KA, Henderson DM, Bian J, Telling GC, Mathiason CK, Hoover EA (2015). Insights into chronic wasting disease and bovine spongiform encephalopathy species barriers by use of real-time conversion. J Virol.

[72] Morris HR, Spillantini MG, Sue CM, Williams-Gray CH (2024). The pathogenesis of Parkinson's disease. Lancet.

[73] Leccese D, Cornacchini S, Nacmias B, Sorbi S, Bessi V (2023). Creutzfeldt-Jakob disease in a patient with previous COVID-19 infection: “the virus caused the derangement in my brain”. J Alzheimers Dis Rep.

[74] Lopez-Font FJ, Vega-Mena CA, Jimenez A, Scharbaai-Vázquez R, García-Santiago E (2025). Post-COVID neurodegeneration: a Puerto Rican case of rapidly fatal Creutzfeldt-Jakob disease. Cureus.

[75] Bernardini A, Gigli GL, Janes F, Pellitteri G, Ciardi C, Fabris M, Valente M (2022). Creutzfeldt-Jakob disease after COVID-19: infection-induced prion protein misfolding? A case report. Prion.

[76] Young MJ, O'Hare M, Matiello M, Schmahmann JD (2020). Creutzfeldt-Jakob disease in a man with COVID-19: SARS-CoV-2-accelerated neurodegeneration?. Brain Behav Immun.

[77] Nasiri E, Naseri A, Yazdchi M (2021). Is there a link between COVID-19 and Creutzfeldt-Jakob disease? A case report. J Res Clin Med.

[78] McGrath A, Pai H, Clack A (2023). The rapid progression of Creutzfeldt-Jakob disease with concomitant COVID-19 infection: a case report. Clin Med (Lond).

[79] Dyachenko AG, Dyachenko PA (2023). Creutzfeldt-Jakob disease in man co-infected with SARS-COV-2-virus, and human herpesvirus type 7. Prev Med Theory Pract.

[80] Albadr F, Aldusaymani SM, Aldobikhi YA, Alkhaldi SI, Sendy HS, Aldosari HS, Aljurayyad AS (2024). Creutzfeldt-Jakob disease in a Saudi female: a case report. Cureus.

[81] Pimentel GA, Guimarães TG, Silva GD, Scaff M (2022). Case report: Neurodegenerative diseases after severe acute respiratory syndrome coronavirus 2 infection, a report of three cases: Creutzfeldt-Jakob disease, rapidly progressive Alzheimer’s disease, and frontotemporal dementia. Front Neurol.

[82] Ciolac D, Racila R, Duarte C (2021). Clinical and radiological deterioration in a case of Creutzfeldt-Jakob disease following SARS-CoV-2 infection: hints to accelerated age-dependent neurodegeneration. Biomedicines.

[83] Perna A, Colaizzo E, Ladogana A, Silvestri G, Baiardi S (2024). Creutzfeldt-Jakob disease in a man surviving COVID-19: disentangling a casual or causal association by neuropathology. Neurol Sci.

[84] Yong CS, Maniam EJ, Chang CW, Lai JY, Ho CS (2023). Case report: Creutzfeldt-Jakob disease presenting with anxiety symptoms in a COVID-19 post-infection patient. Front Neurol.

[85] Makhoul K, Beeber T, Cordero R, Khan A, Saliaj M (2023). Prion disease after COVID-19: a case report. Am J Case Rep.

[86] Tayyebi G, Malakouti SK, Shariati B, Kamalzadeh L (2022). COVID-19-associated encephalitis or Creutzfeldt-Jakob disease: a case report. Neurodegener Dis Manag.

[87] Burakgazi AZ (2025). The onset of Creutzfeldt-Jakob disease after COVID-19: navigating the diagnostic challenges and clinical evolution. Case Rep Neurol Med.

[88] McGrath A, Pai H, Clack A (2023). Rapid progression of probable Creutzfeldt-Jakob disease with concomitant COVID-19 infection. BMJ Case Rep.

[89] Colaizzo E, Prosperini L, Petrucci A (2024). Creutzfeldt-Jakob disease associated with E200K mutation and SARS-CoV-2 infection: pure coincidence or neurodegenerative acceleration?. Clin Transl Neurosci.

[90] Olivo S, Furlanis G, Buoite Stella A, Fabris M, Milanic R, Zanusso G, Manganotti P (2023). Rapidly evolving Creutzfeldt-Jakob disease in COVID-19: from early status epilepticus to fatal outcome. Acta Neurol Belg.

[91] Alloush TK, Alloush AT, Abdelazeem Y, Shokri HM, Abdulghani KO, Elzoghby A (2023). Creutzfeldt-Jakob disease in a post-COVID-19 patient: did SARS-CoV-2 accelerate the neurodegeneration?. Egypt J Neurol Psychiatr Neurosurg.

[92] Singh H, Yura T, Kak V (2023). Sporadic Creutzfeldt-Jakob disease with COVID-19 infection: a case report. Cureus.

